# A Systematic Review of Medicinal Plants of Kenya used in the Management of Bacterial Infections

**DOI:** 10.1155/2022/9089360

**Published:** 2022-03-24

**Authors:** Elizabeth A. Odongo, Peggoty C. Mutai, Beatrice K. Amugune, Nelly N. Mungai

**Affiliations:** ^1^Department of Pharmacology and Pharmacognosy, School of Pharmacy, University of Nairobi, P.O. Box 19676-00202, Nairobi, Kenya; ^2^Department of Pharmaceutical Chemistry, School of Pharmacy, University of Nairobi, P.O. Box 19676-00202, Nairobi, Kenya

## Abstract

Kenya's vision 2030 partly aims at ensuring adequate health care for all, and the integration of traditional healthcare practices into the national healthcare system would present a more rapid alternative towards the realization of universal health coverage in Kenya. Currently, research on Kenyan medicinal plants with potential antibacterial activity remains vastly fragmented across numerous literature studies and databases; thus, it is imperative to collate and appraise these data for the ease of future research and possible clinical application. *Objective*. This review aims at exploring and compiling research evidence on medicinal plants used in the management of bacterial infections in Kenya, with a focus on their efficacy and safety. *Methodology*. A comprehensive web-based systematic review using the Preferred Reporting Items for Systematic Reviews and Meta-Analyses (PRISMA) guidelines was executed to highlight the Kenyan medicinal plants used for the management of bacterial infections in Kenya. This review includes studies published until January 2021 from the PubMed, Science Direct, AJOL, and Google Scholar databases. *Results*. A total of 105 Kenyan medicinal plants belonging to 43 families have their *in vitro* activity against various human pathogenic bacteria evaluated. Plants from the Lamiaceae, Rutaceae, and Fabaceae families were the most commonly studied. *Aloe secundiflora*, *Toddalia asiatica*, *Senna didymobotrya*, *Warbugia ugandensis*, *Tithonia diversifolia*, *Fuerstia africana*, *Olea africana,* and *Harrisonia abyssinica* were the plants frequently evaluated within Kenya. The plants with the strongest antimicrobial activities were *Toddalia asiatica*, *Hagenia abyssinica*, *Ocimum gratissimum*, *Harrisonia abyssinica*, *Senna didymobotrya*, *Olea Africana*, *Camellia sinensis,* and *Tarmarindus indica*. *Conclusion*. Based on a published work, it is evident that traditional medicine is seemingly an acceptable and efficient system among Kenyan communities in the management of bacterial infections. Kenya's rich biodiversity with diverse secondary metabolites presents a promising source of new therapeutic alternatives with possibly different mechanisms of action against bacteria.

## 1. Introduction

Despite the remarkable investment in health care witnessed over the past decade, microbial infections remain a major threat to human and animal health and are a cause of morbidity and mortality especially in low- and middle-income countries (LMICs). The rising cases of antibiotic resistance present a major health problem globally, and there is an immediate need for strategies to manage it as it relentlessly compromises the effectiveness of antimicrobial therapy and increases the threat of therapeutic failure [1–3]. Due to an inefficient antimicrobial resistance (AMR) surveillance system, the exact liability of AMR in Kenya is indefinite although cases such as reduced susceptibility of community-acquired pneumococci, *Vibrio cholera* outbreaks, and methicillin-resistant *Staphylococcus aureus* (MRSA) from hospitalized patients have been reported [4].

Herbalism is the most preferred form of traditional medicine and is highly lucrative in the international market with annual sales ranging from US dollar 5 billion in Western Europe to US dollar 14 billion in China [5]. In Africa, herbal products are available in most markets in the urban centers and rural areas [6]. Irrespective of the accessibility to modern medicines, various communities in Kenya (either deliberately or due to economic limitations) utilize medicinal plants for the management of microbial infections and other diseases; thus, various legislations are actively being formulated to regulate this practice [7]. Presently, there are over 400 plant species used for the management of common diseases in East Africa documented in several ethnobotanical [8–10].

As a developing nation with numerous healthcare challenges such as the high costs of medications, Kenya needs to grow its scientific base and create logical and effective solutions to manage them. Laboratory investigations and various clinical trials have often suggested the positive effects of phytomedicines both *in vivo* and *in vitro*; however, there has been little systematic appraisal of their benefits [11]. Due to their unrivaled chemical diversity, plants offer the infinite potential for innovative and effective antimicrobial agents, but there is the scantiness of information in regard to their efficacy and their safety levels [12]. Critical consideration to the prospect of producing pharmaceutical products using local raw materials is a worthy endeavor to ensure the affordability of drugs. In a bid to provide herbal practitioners and consumers with insight, this study primarily aimed at evaluating the bioactivity of Kenyan medicinal plants useful in the management of bacterial infections.

## 2. Materials and Methods

A comprehensive web-based systematic review using the Preferred Reporting Items for Systematic Reviews and Meta-Analyses (PRISMA) guidelines on identification, screening, eligibility, and inclusion was executed to highlight the medicinal plants used for the management of bacterial infections in Kenya. This review covers published literature from 1994 up to January 2021 obtained from the PubMed, Science Direct, African Journals Online (AJOL), and Google Scholar databases. Grey literature [13] from the local university repositories and conference proceedings were also included in this review [14].

The literature search was performed using search terms identified from previous similar reviews. The Boolean search operators (AND and OR) were used to effectively combine the search terms [15]. The following search terms were used: Kenya AND antimicrobial plants OR Kenyan AND antimicrobial plants. Kenyan AND antibacterial plants OR Kenyan AND antibacterial plants. Kenya AND traditional medicine AND antimicrobial plants OR Kenyan AND traditional medicine AND antimicrobial plants, Kenya AND traditional medicine AND antibacterial plants OR Kenyan AND traditional medicine AND antibacterial plants, Kenya AND ethnopharmacological AND antimicrobial plants OR Kenyan AND ethnopharmacological AND antimicrobial plants and Kenya AND ethnopharmacological AND antibacterial plants OR Kenyan AND ethnopharmacological AND antibacterial plants [16].

Screening of search outputs was performed in two stages. First, the title and abstract of identified journal articles/theses were overviewed based on PICO (Participants Intervention Comparison and Outcomes) and the studies classified as “yes” or “no” based on the information provided by the title and abstract. Thereafter, suitable articles/theses were downloaded and critically assessed for inclusion in the review [16].

The studies eligible for inclusion were limited to the English language. The assessment of eligibility of studies was performed by at least two people, independently, using the Critical Appraisal Skills Programme (CASP) appraisal checklist as a guide [16]. This study excluded research data from papers with poor methodology and retracted studies. The quality of the papers was assessed based on study design, description of the subject, method and assay, variables assessment, control groups, and data collection. To minimize bias, data extraction from selected study reports was independently performed by two reviewers and any disagreements resolved through discussion with the third reviewer [17].

## 3. Results

The study included research data from the pharmacological assays/ethno-medicinal studies reporting on Kenyan medicinal plants used for the treatment of bacterial infections. The initial database search identified a total of 105, 157 articles. After removing the duplicates (*n* = 15000), 89857 studies were excluded based on the title and abstract. Three hundred (300) full-text articles were assessed for eligibility, from which 211 were excluded based on scope, methodological approach, and very little/no bioactivity reported. A total of seventy-nine (79) studies regarding the *in vitro* antibacterial activity of Kenyan medicinal plants were ultimately included in the review. No *in vivo* studies within Kenya on Kenyan medicinal plants with antibacterial activity were found.

Data collected included herbal plant name, plant family, part of plant used for extraction, extraction/preparation method, concentrations of extracts, bacteria species, data on reported activity, toxicity, exposure time, geographical information, the year of publication, and the first author (Table 1). A total of 105 medicinal plants from 43 families were studied for *in vitro* activity against various human pathogenic bacteria. Plants from Lamiaceae, Rutaceae, and Fabaceae families were the most common (Table 1).

*Aloe secundiflora* (5), *Toddalia asiatica* (5), *Senna didymobotrya* (5), *Warbugia ugandensis* (5), *Tithonia diversifolia* (4), *Fuerstia africana* (4), *Olea africana* (4), and *Harrisonia abyssinica* (4) were the plants frequently evaluated within Kenya. The plants with the strongest antimicrobial activities were Toddalia asiatica, *Hagenia abyssinica*, *Ocimum gratissimum*, *Harrisonia abyssinica*, *Conyza sumatrensis*, *Senna didymobotrya*, *Aloe secundiflora*, *Olea Africana*, *Vernonia glabra*, *Camellia sinensis*, *Tetradenia riparia,* and *Tarmarindus indica* as they exhibited high mean inhibition zone values or low minimum inhibitory concentration (MIC) values. The zones of inhibition (ZOIs) were interpreted as low activity (1 mm–6 mm), moderate activity (7 mm–10 mm), high activity (11 mm–15 mm), and very high activity (>16 mm) (Zaidan et al., 2005). The frequently analyzed plant parts were leaves (37%), bark/stem bark (47%), fruits/seeds (5), pods (1%), and roots (24%). Water and methanol were the most used solvents for plant extract preparation, whereas ethanol and dichloromethane were the least utilized solvents used (Table 1).

**Figure 1:**
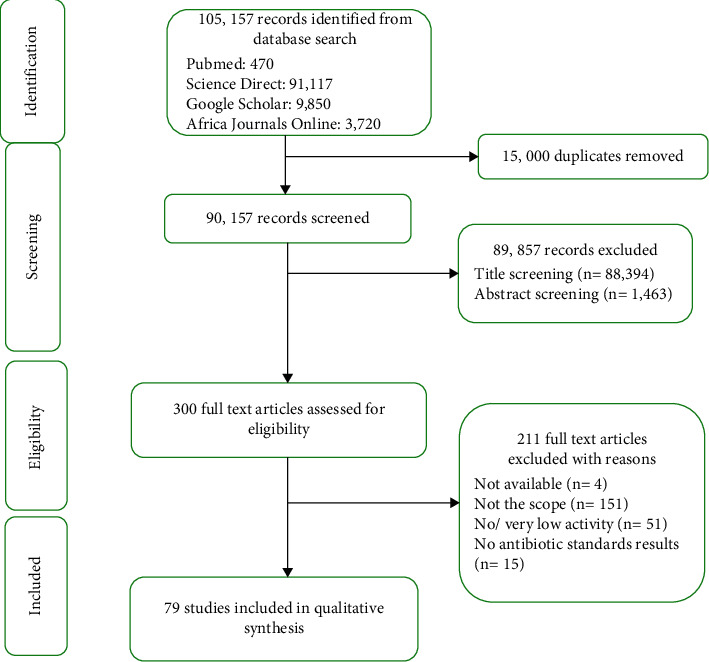
Preferred Reporting Items for Systematic Reviews and Meta-Analyses (PRISMA) statement of search results.

The reported medicinal plants were commonly used in the treatment of STIs, respiratory diseases, diarrhea, and oral infections (Table 1). (Figure 1).

## 4. Discussion

Plants generally accumulate diverse bioactive compounds in varying concentrations in the different parts of a plant, and this eventually affects the efficacy of medicinal plants. The leaves (37%), bark/stem bark (47%), and roots (24%) were the most utilized plant parts against bacterial infections. The variances in their antimicrobial activities could be due to the synergistic or antagonistic actions of various secondary metabolites present [60].

### 4.1. Nutraceuticals

From the review, several common foods/spices are reported to have potential antibacterial benefits. For example, green tea (*Camellia sinensis*) that is often famed for its antioxidant activity, exhibited good antibacterial activity zone of inhibition (ZOI) of 21.3 Ł} 0.33mm against *E.coli* and a ZOI of 22.3 ± 0.50 mm against *S. aureus* compared to gentamicin 22.3 Ł} 0.50 mm (against E. coli) and ZOI 23.7 Ł} 0.33 mm (against *S. aureus*) at a concentration of 0.1 mg/ml [67]. The aqueous crude green tea extracts at a concentration of 400 mg/ml exhibited ZOI of 20 ± 0.0 mm which was similar to that of streptomycin against *S. aureus*. The extract also displayed a ZOI of 18 ± 0.0 mm against E. coli compared to ZOI of 10 ± 0.0 mm of streptomycin against E. coli 20 ± 0.0 mm, ZOI of 18 ± 0.0 mm, and MIC 200 mg/ml against E. coli compared to streptomycin 10 ± 0.0 mm [69]. The Bambara nut (*Vigna subterranea*) had an MIC value of 7.72 ± 0.35 *μ*g/ml for *E. coli*, 12.5 ± 0.32 *μ*g/ml for *S. aureus* and 7.95 ± 0.10 *μ*g/ml for *P. aeruginosa* at 100 *μ*g/ml, and showed zone of inhibition of 27 ± 0.74 mm, 25.3 ± 0.40 mm, and 25.1 ± 0.24 mm *E. coli*, *S. aureus,* and *P. aeruginosa,* respectively [109].

### 4.2. Complementary Medicine

As has been shown in previous ethnomedical surveys by Omwenga et al., traditional medicine is widely practiced in Kenya and is culturally acceptable. It is estimated that about 75% population in Kenya seeks health care among traditional healers [8–10, 150]. In certain instances, people utilize both traditional and modern medicine simultaneously. Njoroge and Kibunga noted that herbal products were used as complementary therapy in the management of diarrhea by residents in Thika, Kenya [151]. The lack of enquiry about Traditional Complementary and Alternative Medicine (TCAM) use and the conventional healthcare providers' negative attitude towards TCAM were cited as some of the reasons why patients fail to reveal their TCAM use [152].

The regulatory framework for the practice of traditional medicine in Kenya is still underway [153], but several crude drugs or formulated herbal products with reported antibacterial activity are already available in the Kenyan market, for example, the Lifebuoy germ protection antibacterial herbal hand and body soap and the Dettol herbal bar soap. Skin care products (soaps and lotions) formulated from plant extracts (Thevetia peruviana, Tithonia diversifolia, Azadirachta indica, Aloe secundiflora) had antimicrobial properties. Soap made from Tithonia diversifolia plant extract was the most effective against *E. coli*, while Azadirachta indica soap was the most effective against C. albicans. T. diversifolia soap exhibited the highest activity against *E. coli* [154].

The ethanolic extract of *E*. *divinorum* root bark had a MIC of 25, 50, and 25 *μ*g/ml for *Streptococcus* pyogenes, *Staphylococcus aureus,* and *Escherichia coli*, respectively (Table 1). A herbal toothpaste formulated with the ethanol extract of *E*. *divinorum* root bark had a higher antimicrobial activity against the tested microorganisms compared to Colgate herbal toothpaste formulated with fluoride [77]. Also, the formulation containing the aqueous extracts of *T. asiatica* (50 mg/ml) stem bark exhibited pronounced antimicrobial activity as indicated by zone of inhibition diameters of 24 mm (MRSA) and 22 mm (M. *gypseum*) compared to 22 mm and 14 mm, respectively, by the commercial hand wash (50 mg/ml). In the model hand washes efficacy experiment, the formulated herbal detergent attained a 78.8% reduction of pathogenic load as compared to 67.9% reduction with the commercial hand wash [37].

Unfortunately, despite the surge in the consumption herbal products and the limited number of standardized herbal products in the Kenyan market, the pharmacovigilance for herbal medicines is nonexistent in Kenya [155]. The increased demand for herbal products has resulted in the market being flooded with adulterated products and false herbal claims on the products' labels for marketing purposes. For instance, 50% of the investigated products by Ngari et al. [75] lacked antimicrobial activity against test bacteria (*Staphylococcus aureus*, *Pseudomonas aeruginosa*, *Proteus vulgaris*, *Bacillus subtilis*, *Candida albicans*, *Escherichia coli*, *Streptococcus mutans*, *Enterococcus faecalis*, and *Lactobacillus acidophilus*). The evaluation of herbal suspensions (used in the management of oral health in Nairobi County, Kenya) by Ngari et al. [75] reported a lack of detectable phytochemicals in the suspensions and noted that this occurrence could be due to very low concentrations of phytochemicals that could not be detected by standard laboratory methods or mineral adulterants might have been used. Studies have demonstrated that antimicrobial properties of natural products can be enhanced by the addition of metal ions [14, 156].

### 4.3. Polyherbalism

The use of herbs as combinations is common practice with many herbal practitioners and aimed at giving better results as compared to single herbs and also treating more than one ailment [157]. Ngari et al. [75] evaluated herbal pastes and suspensions used in the management of oral health in Nairobi County, Kenya, and various products showed significant antimicrobial activity that is comparable to positive control. For example, product HS4 composed of *W. ugandensis*, *M. piperita,* and *S. aromaticum* had ZOI of 33.1 ± 0.85 mm, 20.3 ± 1.71 mm, and 19.3 ± 1.65 mm against *E. coli*, *S. aureus,* and *P. aeruginosa,* respectively, compared to that of co-trimoxazole of 27.5 ± 0.7 mm, 8.5 ± 0.5 mm, and 10 ± 0.0 mm, respectively. Product HS5 composed of *Aloe vera gel*, *W. ugandensis*, and *W. sominifera* had a ZOI of 25.3 ± 0.25 mm, 20.25 ± 1.26 mm, and 21.5 ± 1.71 mm against *E. coli*, *S. aureus,* and *P. aeruginosa,* respectively, compared toco-trimoxazole 27.5 ± 0.7 mm, 8.5 ± 0.5 mm, and 10 ± 0.0 mm, respectively. This biological activity is attributed to the presence of various secondary metabolites in plants [14].

Mbuthia et al. evaluated the synergistic properties of water-soluble green and black tea extracts with penicillin G. The antimicrobial results showed a marked increase in the inhibition zone diameters on the combination of green tea extracts with penicillin G. The catechins, theaflavins, and thearubigins are the antimicrobial agents present in tea [67].4 Synergistic inhibition by green tea extracts and penicillin G is due to the presence of dual binding sites on the bacterial surface for antibiotic and tea extract [158].

### 4.4. Bioactivity/Assay Methods

Disc diffusion method was the preferred method to assay for antibacterial activity. A clearing zone of 9 mm or greater for Gram-positive and Gram-negative bacteria was used as the criterion for designating significant antibacterial activity [159]. The in vitro MIC results were classified as described in the study by Pessini et al., 2003: the antimicrobial activity of the extracts that displayed MIC lower than 100 *μ*g/ml was considered very high; 100–500 *μ*g/ml, high; 500–1000 *μ*g/ml, moderate; 1000–4000 *μ*g/ml, low; and anything above this, inactive. The plants with the strongest antimicrobial activities were Toddalia asiatica, *Hagenia abyssinica*, *Ocimum gratissimum*, *Harrisonia abyssinica*, *Conyza sumatrensis*, *Senna didymobotrya*, *Aloe secundiflora*, *Olea Africana*, *Vernonia glabra*, *Camellia sinensis*, *Tetradenia riparia,* and *Tarmarindus indica* as they exhibited high mean inhibition zone values or low minimum inhibitory concentration (MIC) values.

Several plants exhibited a high activity superior or comparable to the standard antibiotic drugs (Table 1); the methanol-dichloromethane extract (100 mg/ml) of *Harrisonia abyssinica* had a ZOI of 20 ± 1.6 mm compared to gentamycin (ZOI of 18 ± 1.2 mm) against *S. aureus* and a ZOI of 30. ± 1.7 mm against E. coli compared to gentamycin ZOI of 15.1.3 mm against E. coli [41]. The methanolic extract of *Croton macrostachyus* exhibited ZOI (23.66 mm) compared to (21.33 mm) amoxicillin against *S. aureus* and ZOI (18.0 mm) compared to (17.58 mm) amoxicillin against *P. aeruginosa.* The methanolic extract had an MIC of 37.50 mg/ml compared to 18.75 mg/ml cefpodoxime against *S. aureus* and 18.75 mg/ml compared to 9.372 mg/ml cefpodoxime against *P. aeruginosa* [79].

Plants such as *Toddalia asiatica*, *Hagenia abyssinica*, *Senna didymobotrya*, *Aloe secundiflora,* and *Camellia sinensis* displayed good activities; thus, they may be considered for the assessment of *in vivo* activity and possibly formulated into different consumable forms. Korir et al. recommended that for plants with very low or no activity, bioactivity on all parts of the plants, for example, root, stem bark, and leaves combined ought to be done against a wide variety of pathogenic bacteria in order to conclusively report that a certain plant is inactive [29].

### 4.5. Toxicity

Despite herbal remedies being affordable, their lack of efficacy and safety evaluation is a great impediment to their acceptance into mainstream medicine. The safety assessment of herbal remedies remains a challenge as most of the studies of herbal medicines are directed at the toxicological properties of single plant formulations, yet most herbal preparations, especially those used in traditional medicine, contain multiple herbs [160].

From this review, 45% of the plants were relatively safe, 44% of the plants have not been assessed for their safety, and 11% of the plants were reported to have high toxicity (Table 1). The plants with very high toxicity can be further explored for the antitumor activity or as insecticides.

### 4.6. Plant Conservation Status

Other than *W*. *ugandensis* and *Prunus africana*, most of the plants identified in this review are largely available and are not under any serious threat to become extinct. Since most of them are obtained from wild habitats, sustainable use of the reported medicinal plants against bacterial infections is advised as a conservation measure. The cultivation of wild medicinal plants is an important approach to safeguard the herbal industry. Biotechnological techniques such as plant cell or tissue culture, biochemical conversions, and clonal propagation of indigenous medicinal plants are another potential strategy in improving herbal medicine [161].

## 5. Conclusion

This review demonstrates the potential of medicinal plants to treat bacterial infections alongside justifying the use of these plant traditional medicine. It may serve as a starting point of research geared towards the clinical application of these plants. There is a need for standardization to improve the acceptance of herbalism by mainstream health practitioners.

## 6. Recommendation

Further research into the *in vivo* activity of plants displayed remarkable *in vitro* activity. Plants exhibiting strong antibacterial activity can be evaluated for their interactions with conventional antibiotics, and those displaying synergistic activity may provide useful leads in antibiotic therapy.

## Figures and Tables

**Table 1:** Systematic review of Kenyan antibacterial medicinal plants.

Plant	Ethnopharmacological use	Part used	Bioactivity	Assay method used	Collection site in Kenya	Side effects/contraindications/toxicity	References
*Aloe secundiflora* Engl. (Asphodelaceae)	Candidiasis, diarrhea, sore throat, and wound healing	Leaves	The ethanol leaf extract exhibited a ZOI of 17.0 ± 0.8 mm compared to 8.4 ± 0.7 mm (erythromycin) and 8.0 ± 0.8 mm (gentamycin) against *Streptococcus pneumoniae*	Disk diffusion method	Eastern Kenya	None reported	[18]
	Wounds, appetizer, and malaria		The methanol leaf extract (100 mg/ml) exhibited a ZOI of 17 ± 1 mm against *Staphylococcus aureus*, 18 ± 2 mm, *Bacillus subtilis*, 17 ± 2 mm. *K. pneumoniae,* and 19 ± 2 mm against *E. coli*	Agar well assay	Department of Biological Sciences, Egerton University	Not reported	[19]
	Stomachache, polio, malaria, and chest problems		The methanol leaf extract (1 g/ml) exhibited a ZOI (13.0 ± 0.17 mm) compared to (25.0 ± 1.06) ciprofloxacin against *S. aureus*, ZOI (17.0 ± 1.38 mm) compared to (20 ± 2.47 mm) against *E. coli*, and ZOI (18 ± 0.35 mm) compared to (22.0 ± 1.06 mm) ciprofloxacin against *E. faecalis*	Disk diffusion method	Kenyatta University Arboretum	Not reported	[20]
	Stomachache		The methanol leaf extract had an MIC of 9.375 mg/mL against *P. aeruginosa* compared to amoxicillin 4.687 mg/mL, MIC of 18.75 mg/mL compared to amoxicillin 4.687 mg/mL against *E. coli* (MIC and MBC of 37.5 mg/mL compared to amoxicillin 4.687 mg/mL against *S. aureus* and *S. typhi)*	Broth dilution method	Lake Victoria Region of Kenya	Not reported	[21]
	Wound healing		The methanol leaf extract had an MIC (mg/ml) of 9.1 and an MBC (mg/ml) of 10.4 and exhibited a ZOI of 16 ± 1.27 mm against *E. coli* compared to ciprofloxacin 17 ± 1.38 mm	Disc diffusion method/broth dilution method	Kenyatta University Arboretum	Not reported	[22]
*Tithonia diversifolia* (Hemsl.) A. Gray (Asteraceae)	Constipation, stomach pains, liver pains, indigestion and sore throats and as an antiviral	Leaves	The ethyl acetate leaf extract exhibited a ZOI of 8.0 ± 0.5 mm against *Streptococcus pneumoniae*, compared to 2.4 ± 0.6 mm (gentamycin) 2.2 ± 0.4 mm (erythromycin)	Disk diffusion method	Eastern Kenya	A 70% ethanol extract of the aerial parts was toxic to the kidney and liver toxicity at the lowest dose tested (400 mg/kg). *T. diversifolia* should be used with caution as it may be toxic especially in prolonged use at higher doses	[18, 23]
	Diarrhea		The methanol leaf extract (1 g/ml) exhibited ZOI of 21.6 mm, 19.3, and 18.0 against *S. aureus*, *P. aeruginosa*, and *K. pneumoniae* compared to amoxicillin 23.0 mm, 17.3 mm, and 17. 66 mm, respectively, MIC of 37.5 mg/ml against *S. aureus*	Agar disc diffusion method/broth dilution	Twiga Region in Central Province		[24]
	Skin infections		The ethyl acetate leaf extract exhibited a ZOI of 18.2 mm against *S. typhi* compared to chloramphenicol with a ZOI of 23.3 mm and ciprofloxacin with a ZOI of 26.0 mm	Disc diffusion method	Nyamira County		[25]
	Gastrointestinal disorders		The dichloromethane leaf extract (25 mg/mL) exhibited a ZOI of 18 mm against *S. aureus* and 14 mm against *P. aeruginosa*	Agar well diffusion method	University of Kabianga Botanical Garden, Kericho County		[26]
*Senna didymobotrya* (Fresen.) Irwin & Barneby (Caesalpiniaceae)	Skin diseases, diarrhea, dysentery, laxative, malaria	Roots, Stem barks, leaves	The methanol root extracts exhibited a ZOI of 1.58 cm compared to streptomycin (1.30 cm) against *S. aureus*	Disk diffusion method	Kibuye, Kisumu County	The methanol and dichloromethane crude root extracts of had an LD_50_ of 1927 mg/kg after a period of 14 days. the extracts at high concentration and at a high dose tend to be toxic	[27, 28]
	Malaria, skin conditions, livestock infections		The methanol stem bark extracts (100 mg/ml) had a ZOI of 19.0 mm compared to 30 *μ*g/ml gentamycin (19.0 mm) against *S. aureus*, ZOI (11.0 mm) compared to 30 *μ*g/ml gentamycin (9.0 mm) against MRSA, ZOI (12.0 mm) compared to 30 *μ*g/ml gentamycin (17.0 mm) against *K. pneumoniae*	Disk diffusion method	Bomet District		[29]
	Diarrhea		The methanol leaf extracts (1 g/ml) had ZOI (16.0 mm) compared to (60.0 mm) gentamycin (10 *μ*g/ml) against *B*. *subtilis*. The methanol extracts (1 g/ml) had ZOI (16.0 mm) compared to (24.0 mm) gentamycin (10 *μ*g/ml) against *S. aureus*	Disk diffusion method	Rarieda		[30]
	Diarrhea, fevers, abscesses of the skeletal muscles, and venereal diseases		The methanol 2.5% root bark extract and 7.5% stem bark extracts here inhibited the growth of *S. aureus,* which was also observed in streptomycin (1 g/L)	The area under disease progress stairs (AUDPS)	Siaya, Nakuru, and Nandi counties		[31]
	Oral infections		The ethanol leaf extract (1 mg/mL) exhibited a ZOI of 21.70 ± 0.88 mm, against *P. gingivalis* and (MIC 0.13 ± 0.00 mg/mL and MBC 0.50 ± 0.00 mg/mL). Amoxicillin had a ZOI of 40.3 mm	Agar well diffusion assay	Borabu Sub-county in Nyamira County		[32]
*Toddalia asiatica* L. (Rutaceae)	Food poisoning, malaria, and sore throat	Fruits, stems, barks, roots, leaves	The essential oil (10 *μ*L) exhibited a ZOI (mm) of 21.00 ± 2.08 against *E.coli*, 22.33 ± 1.67 against MRSA and 19.00 ± 1.16 *S. aureus* compared to tetracycline 26.00 ± 0.58 against *E.coli*, 9.00 ± 0.58 against *MRSA* and 11.67 ± 0.88 *S. aureus*	Disc diffusion method	Maseno area, Kisumu County	The root extract showed LD_50_ >1000 mg/kg and CC_50_ >100 *μ*g/ml	[33, 34]
	Malaria and diuretic		The stem bark methanol extract (1 g/ml) had a ZOI of 16.67 ± 0.67 mm against *S. aureus* compared to gentamycin (1.0 *μ*g/disc) 25.33 ± 0.67	Disc diffusion method	Kakamega Forest		[35]
	TB and measles		The methanol root extract exhibited a ZOI (mm) of 7.0 against *E*.*coli*, 6.33 against *S. typhi,* and 8.66 against *S. aureus* compared to tetracycline 20.22 against *E.coli*, 16.00 against *S. typhi,* and 21.33 *S. aureus*. MIC and MBC of 9.375 mg/mL against *S. typhi* and *S. aureus*	Agar disc diffusion (DD) method broth microdilution technique	Bondo (Alego)		[36]
	Skin infections, bronchial pains, and stomachache		A formulated antiseptic herbal detergent exhibited ZOI of 24.30 ± 0.67 mm, 18.00 ± 0.58 mm, 16.00 ± 0.58 and 19.67 ± 0.67 mm against MRSA, *P. aeruginosa*. *E.coli,* and *S. typhi,* respectively, compared to the commercial hand wash 21.67 ± 0.33 mm, 19.67 ± 0.67 mm, 13.67 ± 0.33 mm and 18.33 ± 0.33	Disc diffusion method	Slopes of Kajulu Hills, Lake Victoria Basin		[37]
	Malaria and flu		The stem bark DMSO extract exhibited an ZOI of 10 ± 0.3 mm compared to flucloxacillin 10 ± 0.1 mm against MRSA	Agar well diffusion method	Narok		[38]
*Harrisonia abyssinica* Oliv. (Simaroubaceae)	Stomachache, abdominal pains, fever, nausea, vomiting, plague, swollen testicles, dysentery, gonorrhea, tuberculosis	Whole plant, leaves, barks, berries	The methanol whole plant extract had MIC (6.25 mg/ml) compared to (>1 mg/ml for antibiotic standards) against *S. aureus* and *P. aeruginosa* and MIC (250 mg/ml) against *E. coli*	Broth dilution method	Meru Central District	The methanol root bark extract had LC_50_ (*μ*g/ml) of 198.498 and was considered cytotoxic	[39, 40]
	Pneumonia, malaria, and eye ointment		The methanol leaf extract had an MIC of 100, 15.6, 75, 150 mg/mL against *S. aureus B. cereus*, *P. aeruginosa,* and *E. coli,* respectively, compared to that of 0, 0, 0.25 , 0.25 mg/mL of streptomycin against *S. aureus B*. *cereus*, *P. aeruginosa,* and *E. coli,* respectively, and benzylpenicillin 0.6, 0.6 against *S. aureus* and *B. cereus*	Broth dilution method	Machakos and Kitui		[39]
	Fever, tuberculosis, and snake bite		The methanol-dichloromethane extract (100 mg/ml) had an ZOI of 20.1.6 mm compared to (18.1.2 mm) gentamycin against *S. aureus* methanol-dichloromethane extract (100 mg/ml) had an ZOI 30.1.7 mm compared to (15.1.3 mm) gentamycin against *E. coli*	Agar diffusion assay	Bondo District in Nyanza Province		[41]
	Infertility, menstrual problems, and stomach pain menstrual		The crude extracts showed a moderate activity against *S. aureus* (11 mm), *B. subtilis* (7.8 mm), *P. aeruginosa* (7.0 mm), *E. coli* (8.5 mm)	Disc diffusion method	Chuka, Meru-South District, Tharaka Nithi County		[42]
*Fuerstia africana* T. C. E. Fr. (Lamiaceae)	Urinary problems, tongue infections, diarrhea, skin infections	Leaves, aerial parts	The methanol leaf extracts (1 g/ml) exhibited a ZOI of 17.0 mm compared to (26.0 mm) gentamycin (10 *μ*g/ml) against *B*. *subtilis*. The methanol extracts (1 g/ml) had a ZOI of 19.0 mm compared to (24.0 mm) gentamycin (10 *μ*g/ml) against *S.*. *aureus*, methanol extracts (1 g/ml) had a ZOI of 20.0 mm compared to (26.0 mm) gentamycin (10 *μ*g/ml) against MRSA	Disk diffusion method	Kisii south	Extracts were found to be safe at 5000 mg/kg body weight per day. median lethal dose (LD_50_) of methanol and DCM extracts is >5000 mg/kg	[30, 43]
	Eye ailments, toothache		The hexane leaf extract (100 mg/ml) exhibited a ZOI of 10.67 ± 0.33 mm compared to (17.33 ± 0.33) chloramphenicol (30 *μ*g/ml) against *S. aureus*, ZOI (10.50 ± 0.29 mm) compared to (15.00 ± 0.00) chloramphenicol (30 *μ*g/ml) against MRSA, and ZOI (9.67 ± 0.33 mm) compared to (16.50 ± 0.29) chloramphenicol (30 *μ*g) against *P. aeruginosa*	Agar well diffusion method	Olenguruone, Nakuru County, and Cheptenye, Kericho County		[43]
	Boils		The methanol extract exhibited ZOI of (17.21 ± 0.22) compared to gentamycin (23.88 ± 0.01) against *K. pneumoniae*, ZOI of (14.24 ± 0.35) compared to gentamycin (23.88 ± 0.01) against *E. coli* and ZOI of (15.18 ± 0.42) compared to gentamycin (25.9 ± 0.01) against *S. aureus*	Agar well diffusion	Magadi, Kajiado District of Kenya		[44]
	Oral infections		The chloroform extract exhibited ZOI (15.88 ± 0.54) compared to chloramphenicol (21.7 ± 0.11) against *S. aureus*	Agar well diffusion	Vihiga County, Western Kenya		[45]
*Olea africana* (Oleaceae)	Sore throat and urinary tract infections	Stem bark, barks, twigs, leaves	The ethanol stem bark extract (1 g/ml) exhibited ZOI (18.5 mm) compared to gentamycin (10 *μ*g/ml) 19.5 mm and a MIC of (62.5 mg/ml) against *S*. *aureus*. The methanol extract had a ZOI of 8.3 mm) compared to gentamycin (19 mm) against *E. coli* and ZOI of 9.8 mm compared to gentamycin (21.0 mm) against *P. aeruginosa*	Agar well diffusion/broth dilution	Bomet District	The methanol leaf extract had an LD_50_ value of 3475 mg/kg was; thus, it is nontoxic	[46, 47]
	Sap used for bone setting (fracture)		The aqueous bark extract (1 g/ml) exhibited a ZOI of 10.2 ± 0.6 mm compared to (18.0 ± 0.1) streptomycin (25 *μ*g/ml) against *S. aureus*	Disk diffusion method	Mbeere, and Embu-Eastern Province		[48]
	Chewing stick		The methanol extract exhibited a ZOI of 12.4 mm against *S. aureus*, MIC of 1.5 mg/ml against *E.coli* and 0.30 mg/ml against *S. aureus*	Broth dilution method	University of Kabianga Botanical Garden, Kericho County		[49]
	Chewing stick		The methanol leaf extract (25 mg/mL) exhibited ZOI 18 m against *Pseudomonas aeruginosa*, ZOI 19.20 mm against *S. aureus* and 17 mm against *E. coli*	Broth dilution method	University of Kabianga Botanical Garden, Kericho County		[26]
*Carissa edulis* Vahl. (Apocynaceae)	Malaise, antiviral, and appetizer	Roots, stem, leaves	The ethanol root extract exhibited a ZOI of 8.0 ± 0.9 mm against *S. pneumoniae* compared to 7.2 ± 0.1 mm (gentamycin) and 7.8 ± 0.3 mm (erythromycin)	Disk diffusion method	Eastern Kenya	The oral LD_50_ of the extract was estimated to be >5000 mg/kg. generally safe at doses lower than 1000 mg/kg is in rats ()	[18, 50]
	Kidney problems, pneumonia		MICs and MBCs of 37.50 mg/ml against *S. typhi*	Broth dilution	Transmara West		[51]
	Gonorrhea, asthma		The methanol extract exhibited a ZOI of 9.00 mm against *S*. *typhi* compared to amoxicillin 16.0 mm and both MIC and MBC of 37.5 mg/mL against *S*. *typhi* and *S. aureus*	Agar disc diffusion method/broth microdilution technique	Lake Victoria Region		[36]
*Rhus natalensis Bernh*. (Anacardiaceae)	Malaria	Roots, stems, barks and leaves	The methanol root extract had a MIC of 6.25 mg/against both *S. aureus* and *P. aeruginosa* and had a moderate activity with inhibition zone diameters of 11.6 mm against *S. aureus* and *P. aeruginosa* compared to gentamycin 25.3 mm and 18 mm	Broth dilution method	Kilifi district	The extracts were safe to the mammalian cells	[52]
	Diarrhea and stomachache		The isolated compound (1)-epicatechin exhibited a ZOI of 15 ± 0.3 mm against *S. aureus* and (10 ± 0.2 mm) against *P. aeruginosa* compared to streptomycin 10 *μ*g/disc 22 ± 0.2 mm and 20 ± 0.3 mm, respectively	Disc diffusion method	Kapkonga Iten, Eldoret town		[53]
	Microbial infections		The isolated compound 1 had a ZOI of exhibited ZOI of 21 mm against *S. aureus* compared to Chloramphenicol 20 mm	Agar diffusion method	Thika River in Gatanga division, Central Kenya		[54]
*38*. *Prunus africana* (*Hoolh f.*) *Kalkman* (Rosaceae)	Arrow poisoning and gonorrhea	Barks, stems	The methanol bark extract showed a moderate activity against *S. aureus* (11.0 mm), *B*. *subtilis* (10.7 mm), *P. aeruginosa* (9.7 mm), and *E. coli* (8.0 mm)	Disc diffusion method	Chuka, Meru-South District, Tharaka Nithi County	The bark had an LD_50_ of 2201 mg/ kg. The stem bark extract was determined to be nontoxic at the therapeutic dose of 500 mg/kg body weight	[42, 55]
	Diarrhea		The methanol stem bark extract exhibited a ZOI of 20 mm against *S. aureus* and MIC of 0.073 mg/ml. ZOI of 17 mm with MIC of 0.156 mg/ml against MRSA. ZOI of 15 mm and the MIC of 0.3125 mg/ml against *P. aeruginosa*. . ZOI of 12 mm and the MIC of 2.50 mg/ml against *S. pneumoniae*	Disc diffusion assay	Rift Valley Province of Kenya		[56, 57]
	Chest pain and stomach problems		The hydro-methanolic bark extract exhibited a ZOI of 17.33 ± 0.882 mm against *S. typhi* and ZOI of 12.33 ± 0.333 mm against *Escherichia coli* compared to penicillin 27.67 ± 1.2 mm and 20.33 ± 0.333 mm	Agar well diffusion method	University of Eastern Africa, Baraton		[58]
*Warbugia ugandensis* Sprague (*Canellaceae*)	Diarrhea, constipation and cough,	Bark, roots, leaves, stem bark	The methanol extract (100 mg/ml) exhibited a ZOI of 15.0 mm compared to (18.0 mm) chloramphenicol against *S. aureus*, ZOI (14.0 mm) compared to (24.0 mm) chloramphenicol against MRSA. The Dichloromethane extract had a MIC of 3.125 mg/ml against *S. aureus* and MRSA	Disc diffusion test/broth dilution	Ngong Forest	The extract had a LD_50_ > 5000 mg/kg body weight. Extract displayed no apparent deleterious toxicity	[55, 59]
	STIs, diarrhea, and bronchitis		The methanol extract exhibited ZOI of 3.169 ± 0.27 mg/ml against *S. aureus*	Disk diffusion method	Rift Valley		[60]
	Microbial infections		The methanol extract exhibited a ZOI of 19.33 ± 0.333 mm against *S. epidermidis* compared to penicillin 26.67 ± 0.333, ZOI 17.00 ± 0.882 mm against *B*. *cereus* and ZOI 11.67 ± 0.333 mm against *E. coli* compared to penicillin 31.33 ± 0.333	Disc diffusion	Natural Forest around the University of Eastern Africa, Baraton		[61]
	Hepatitis, gonorrhea tuberculosis, bronchitis, and pneumonia		The stem bark extract of hexane exhibited a ZOI of 11.32 mm against *S. typhi* compared to chloramphenicol 23.3 mm and ciprofloxacin 26.0 mm	Disc diffusion test	Nyamira county		[25]
	Sexually transmitted diseases, throat, and chest infections, diarrhea, and wounds/ulcers		The methanol extracts exhibited a ZOI of 30 mm, 28 mm, and 16 mm for the root, stem‐bark, and leaf extracts, respectively, at 100 *μ*g/ml concentration against *E. coli*. The ZOI for the water extracts was 20 mm, 18 mm, and 12 mm for the root, stem‐bark, and leaf extracts, respectively, at an equivalent concentration of 100 *μ*g/ml compared to Norfloxacin 23 mm. The extracts had an effective MIC of 42 *μ*g/ml	Disc diffusion method/broth dilution	Jomo Kenyatta University of Agricultural and Technology (JKUAT) Botanical Garden		[62]
*Allium sativum* L. (Liliaceae)	Dysentery, spice	Rhizome, bulbs	Garlic juice exhibited a ZOI of 10.0 mm against *P. aeruginosa*, 11.7 mm against *E. coli*, 14.7 mm against *S. aureus* and 17.7 mm for *S. typhi*. The activity of ampicillin on *E. coli* and *S. typhi* was 11.7 mm and 18.7 mm ()	Disc diffusion test	Githurai Market, Nairobi	The LD_50_ was found to be 3034 mg/kg, and maximum tolerated dose was 2200 mg/kg	[63, 64]
	Infection, colds		Garlic extract (GE) 200 *μ*l/ml/ exhibited a ZOI of 14 mm compared to gentamycin 24 mm against *S. aureus*	Disc diffusion method	Nakuru Municipal Council Market in Nakuru Town		[65]
	Reduce blood lipids and blood pressure		The methanolic extract of garlic was effective *against E. coli*, *Staphylococcus aureus,* and *Pseudomonas aeruginosa*, with ZOI of 21 mm, 27 mm, and 28 mm, compared to tetracycline 19 mm, 22 mm, and 27 mm, respectively. The garlic methanolic (GM) extract had 0.14 *μ*g/ml against *S. aureus* and *P. aeruginosa*	Agar well diffusion method/broth dilution	Kenyatta University		[66]
*Camellia sinensis* L. (Theaceae)	Beverage	Leaves	Green tea (0.1 mg/ml) exhibited a ZOI of 21.3 ± 0.33 mm against *E.coli* compared to gentamicin 22.3 ± 0.50 mm and ZOI of 23.7 ± 0.33 mm against *S. aureus* compared to gentamicin 23.2 ± 0.28 mm	Agar well diffusion method	Tea Research Foundation, Kangaita Substation in Kirinyaga	There were no observed adverse effects at 2500 mg/kg body weight/day	[67, 68]
			The aqueous crude green tea extracts 400 mg/ml had a ZOI of 20 ± 0.0 mm against *S. aureus* and MIC 100 mg/ml compared to streptomycin 20 ± 0.0 mm, ZOI of 18 ± 0.0 mm and MIC of 200 mg/ml against *E. coli* compared to streptomycin 10 ± 0.0 mm	Agar well diffusion method	Ngere in Murang'a County		[69]
*Azadirachta indica* A. Juss. (Meliaceae)	Udder infections	Leaves, barks, seeds	The neem extract (NE) 200 *μ*l/ml exhibited ZOI 11 mm against *S. aureus* compared to gentamycin 24 mm	Disc diffusion method	Kisauni in Mombasa County	Not reported	[65]
			Methanol bark extract exhibited ZOI of 25 mm, 24 mm, and 20 mm against *E. coli*, *P. aeruginosa,* and *S. aureus,* respectively, with MIC (mg/ml) of 15, 17, and 16	Disc diffusion method	Chumani in Kilifi North Constituency		[70]
*Tagetes minuta* L. (Asteraceae)	Intestinal disorders and stomach problems	Leaves	The methanolic leaf extract exhibited a ZOI of 17 ± 1.94 mm against *S*. *aureus* compared to vancomycin 25.0 mm and ciprofloxacin 22.0 mm (MIC 8.9 mg/ml; MBC 10.0 mg/ml)	Disc diffusion test/broth dilution	Kenyatta university arboretum		[20, 71]
	Oils may cause irritation to the skin		The methanol extract had a MIC (mg/ml) 8.7 and MBC (mg/ml) 10 and ZOI of 16 ± 1.27 mm against *E. coli* compared to ciprofloxacin 20 ± 3.11 mm	Disc diffusion method/broth dilution method	Kenyatta University Arboretum		[22]
*Adansonia digitata L.* (Bombacaceae)	Opthalmia	Leaves, barks	The synthesized AgNPs exhibited a ZOI of 17.1 ± 0.130 mm against *E*. *coli* and 12.9 ± 0.082 mm against *S. aureus* compared to ciprofloxacin with a ZOI of 33.4 ± 0.443 and 12.9 ± 0.082 mm against *E*. *coli* and *S. aureus*	Disc diffusion technique.	Makueni County	The stem extracts are non-toxic to brine shrimp larvae	[72, 73]
	Diarrhea, dysentery		The organic extract at 200 mg/ml and 100 mg/ml showed the highest inhibition zones of 14.33 mm and 12 mm, respectively, against *MRSA* compared to gentamycin 15.5 mm	Disc diffusion technique	Msambweni District		[74]
*Euclea divinorum Hern* (*Ebenaceae*)	Toothbrush, constipation and ulcers	Stems, barks, leaves	The DCM stem bark extract exhibited ZOI (mm) of 10.8 ± 0.26 against *P. aeruginosa* and 17.0 ± 0.42 against *S. aureus* compared to Augmentin 10.0 ± 0.02 against *P. aeruginosa* and 27.0 ± 0.02 against *S. aureus*	Disc diffusion method	Bunyala (Budalang‟i) district of Busia County	The root extracts have toxic effects and should be used with care; gargling of extracts is recommended instead of swallowing	[75, 76]
	Dental caries		The ethanolic root bark extract had MIC of 25, 50, 25 and 25 *μ*g/ml for *S. pyogenes*, *S. aureus*, *E. coli*	Broth dilution	Elgeyo Marakwet, Rift Valley		[77]
*Salvadora persica L. var. persica* (Salvadoraceae)	Chest problems, stomachache, teeth problems	Roots, stems, barks	The organic root extract had 10 GUs (numerical growth units) at 0.5 mg/ml against *M*. *tuberculosis* compared to Isoniazid that had zero GUs at 0.5 mg/ml	BACTEC mgIT™ 960 system	Various conservancies in Samburu	High concentrations >5 g/kg of the mammal's body weight can result in toxicity	[36, 78]
	Oral thrush and diarrhea		The methanol bark extract exhibited a ZOI of 21.66 mm against *S. aureus* compared to Amoxicillin 21.3 mm. ZOI of 20 mm against *P. aeruginosa* compared to amoxicillin 14.33 mm. ZOI of 15 mm against *E. coli* compared to amoxicillin 23.6 mm	Agar disk diffusion technique	Nkaroni, Wamba Division, Samburu District		[79]
*Plectranthus barbatus Andrews* Lamiaceae)	Oral and throat infections	Leaves, roots	The DCM: MeOH crude leaf extract 200 mg/ml and 100 mg/ml exhibited ZOI (mm) of 13 and 10.3, respectively, against MRSA compared to 14 mm for amoxicillin [50 mg/ml]. The lowest MIC values were observed in DCM fraction (40 mg/ml) against MRSA	Disc diffusion technique/broth dilution technique	Various geographical regions of Kenya	Not reported	[80]
	Stomachache and wounds		The root extracts exhibited a ZOI of 18.67 mm, 20.00 mm, and 25.33 mm in *S. aureus*, MRSA, and *B. cereus* compared to streptomycin 39.67 ± 1.76 mm, 39.67 ± 1.76 mm, and 33.00 ± 1.15 mm ()	Agar well diffusion method	Msambweni Subcounty, Kwale County		[81]
*Cordia purpurea* (*Picc.*) *Aiton* (Fabaceae)	Diarrhea	Roots, barks	The methanolic root extract exhibited a ZOI of 15 mm compared to (21.33 mm) amoxicillin against *S. aureus* and ZOI (23.66 mm) compared to (17.58 mm) amoxicillin against *P. aeruginosa*. The methanolic extract had a MIC of 18.75 mg/ml compared to 18.75 mg/ml cefpodoxime against *S. aureus* and 18.75 mg/ml compared to 9.372 mg/ml cefpodoxime against P. *aeruginosa*	Agar disc diffusion method/broth dilution	Samburu‐Wamba conservancies	Not reported	[79]
			The methanolic extract exhibited a ZOI of 14.33 mm compared to (21.33 mm) amoxicillin against S. aureus and ZOI (19.66 mm) compared to (17.58 mm) amoxicillin against P. aeruginosa. The methanolic extract had MIC of 37.50 mg/ml compared to 18.75 mg/ml cefpodoxime against S. aureus and 37.50 mg/ml compared to 9.372 mg/ml cefpodoxime against P. aeruginosa	Agar disc diffusion method/broth dilution	Samburu‐Wamba conservancies		[79]
			Organic bark extract exhibited zero GUs at 0.5 mg/ml against M. tuberculosis and *M. kansasii* compared to Isoniazid that had zero GUs at 0.5 mg/ml	BACTEC mgIT™ 960 system	Various conservancies in Samburu		[36]
*Croton macrostachyus* Hochst. Ex Delile: (Euphorbiaceae)	Diarrhea, stomach ache	Barks, roots	The ethyl acetate bark extract exhibited ZOI between 10.1 ± 0.6 mm and 16.0 ± 1.2 mm against *S. typhi*, *E. coli* and *K. pneumoniae*	Agar disc diffusion method	Baraton Community in Nandi District of Kenya	The aqueous stem extract does not provoke death until the dose 16 g/kg. There is a wide margin of safety for the therapeutic use of the extract	[82, 83]
			The methanolic extract exhibited a ZOI of 23.66 mm compared to (21.33 mm) amoxicillin against *S. aureus* and ZOI (18.0 mm) compared to (17.58 mm) amoxicillin against *P. aeruginosa.* The methanolic extract had an MIC of 37.50 mg/ml compared to 18.75 mg/ml cefpodoxime against S. aureus and 18.75 mg/ml compared to 9.372 mg/ml cefpodoxime against P. aeruginosa	Agar disc diffusion method/broth dilution	Samburu‐Wamba conservancies		[79]
*Ocimum gratissimum L.* (Lamiaceae)	Ear infections, tooth gargle	Leaves	The essential oil from leaves exhibited ZOI (26.6 ± 5.7 mm) compared to (24.5 ± 0.7 mm) chloramphenicol against *S. aureus*. ZOI (21.7 ± 2.1 mm) compared to (32.5 ± 2.5 mm) chloramphenicol against *E. coli*	Agar disc diffusion method	Meru	The oil can cause an inflammatory response	[84, 85]
	Sore eyes and rectal prolapse		The essential oil from leaves extract had ZOI (21.7 ± 2.1 mm) compared to (28.0 ± 07 mm) chloramphenicol against *E. coli*. ZOI (26.6 ± 5.7 mm) compared to (23.5 ± 2.1 mm) chloramphenicol against *S. aureus*	Agar disc diffusion method	Meru District of Eastern Kenya		[86]
*Ocimum suave* Wild (Lamiaceae)	Ear infections, cough and disinfectant	Leaves, roots	The methanolic leaf extract had a MIC of (6.25 mg/ml) compared to >1 mg/ml for antibiotic standards against *S. aureus* and methanolic extract had an MIC of 31.25 mg/ml compared to >1 mg/ml for antibiotic standards against *P. aeruginosa* and *E. coli*	Broth dilution method	Meru Central district	The aqueous leaf extract is nontoxic in acute and subchronic intake. No gross abnormalities, teratogenic, or histological changes observed	[40, 87]
	Stomach ache		The methanol root extract exhibited a mean ZOI of 14 mm against *S. aureus* compared to amoxicillin 21.3 mm, ZOI of 21 mm against *P. aeruginosa* compared to amoxicillin 17.5 mm and ZOI of 18 mm against *E. coli* compared to amoxicillin 23.6 mm	Agar disk diffusion technique	Namunyak, Wamba division, Samburu district		[79]
*Premna resinosa* (*Hochst.*) *Schauer* (Compositae)	Respiratory-related illnesses	Roots	Dichloromethane root extract had a MIC of 31.25 *μ*g/ml against MRSA, while ethyl acetate fraction had a ZOI of 22.3 ± 0.3 against *S. aureus* compared to 33.7 ± 0.3 mm (oxacillin 10 *μ*g/disc and gentamycin 10 *μ*g), methanolic extract had a ZOI of 8.7 mm compared to (22 mm) oxacillin 10 *μ*g/disc and Gentamycin 10 *μ*g against *S. aureus* and ZOI 11.7 mm) compared to (24 mm) oxacillin 10 *μ*g/disc and gentamycin 10 *μ*g against *E. coli*	Disc diffusion and microdilution techniques	Mbeere community, Kenya	The dichloromethane and ethyl acetate fractions were within the acceptable toxicity limit (CC_50_ < 90)	[88]
*Hagenia abyssinica* (Bruce) JF Gmel (Rosaceae)	Diarrhea, stomachache, tongue infections, sores	Stem bark, leaves	The dichloromethane/methanol stem bark extract exhibited ZOI of 19.0 mm against *S. aureus* compared to Erythromycin (0.01 mg/ml) 22 mm, ZOI of 20.0 mm against *E. coli* compared to Erythromycin 0.01 mg/ml 20.0 mm, ZOI of 18 mm against *B. subtilis* compared to Erythromycin 0.01 mg/ml 20 mm	Agar well diffusion method	Aberdare ranges, Kiburu Forest Station	The extracts were safe at 5000 mg/kg body weight per day. Median lethal dose (LD_50_) of methanol and DCM extracts is >5000 mg/kg	[43, 89]
			The hexane leaf extract (100 mg/ml) had a ZOI of 16.67 ± 0.67 mm compared to (17.33 ± 0.33) chloramphenicol (30 *μ*g/ml) against *S. aureus*, ZOI (19.33 ± 1.33 mm) compared to (15.00 ± 0.00) chloramphenicol (30 *μ*g/ml) against MRSA and ZOI (13.00 ± 1.00 mm) compared to (16.50 ± 0.29) chloramphenicol (30 *μ*g) against *P. aeruginosa*	Agar well diffusion method	Olenguruone in Nakuru County and Cheptenye in Kericho County		[43]
*Clerodendrum myricoides* (Hochst.) R. Br. ex Vatke: (Lamiaceae)	Respiratory diseases, tonsillitis, eye infections, gonorrhea	Whole plant	The methanol extracts (1 g/ml) had a ZOI of 14.7 ± 0.3 mm compared to (17.0 mm) gentamycin (10 *μ*g/ml) against *E. coli* and ZOI (20.3 ± 0.3 mm) compared to (33.0 mm) gentamycin (10 *μ*g/ml) against *S*. *aureus*	Disk diffusion method	Mbeere Community, Kenya	The methanol extracts within the acceptable toxicity limit with a CC_50_ of >500 *μ*g/ml) the LD_50_ value of 3475 mg/kg and thus is non-toxic	[90]
	Pneumonia		The aqueous extract (1 g/ml) exhibited ZOI (13.8 ± 0.2 mm) compared to (18.0 ± 0.1) streptomycin (25 *μ*g/ml) against *S. aureus*	Disk diffusion method	Mbeere, and Embu-Eastern Province		[48]
*Securidaca longipedunculata* Var. parvifolia (Polygalaceae)	Infusion reduces swellings	Roots, barks	The aqueous extract (1 g/ml) had a ZOI of 12.5 ± 2.2 mm compared to (18.0 ± 0.1) streptomycin (25 *μ*g/ml) against *S. aureus*	Disk diffusion method	Mbeere, and Embu-Eastern Province	The extract has an LD_50_ value of 771 mg/kg body weight and is nontoxic at relatively high concentrations	[48, 91]
	Sexually transmitted infections		The bark and root extract exhibited ZOI of 20.1 mm and 13.5 mm, respectively, against *N. gonorrhoeae*, compared to 12 mm and 19.1 mm ciprofloxacin and tetracycline	Disc diffusion method	Bungoma County		[92]
*Tarmarindus indica* L. (Fabaceae)	Meat preservative	Fruit paste, bark	The water extract exhibited a ZOI of 34.67 mm, and 24 mm against *E. coli* and *S. aureus,* respectively, compared to chloramphenicol 16 mm and 18 mm	Disc diffusion test	Chepararia and Kongelai subcounties of West Pokot County	The pulp extract of *Tamarindus indica* at 3000 mg/kg and 5000 mg/kg body weight of resulted in no mortality and is practically nontoxic	[93, 94]
	Diarrhea, typhoid	Bark	The methanol bark extract (1 g/ml) had a ZOI of 14.5 mm compared to (24.0 mm) gentamycin (10 *μ*g/ml) against *S. aureus*	Disk diffusion method	Rarieda		[30]
*Zanthoxylum chalybeum Engl.* (*Rutaceae*)	Malaria, pneumonia, sore throat	Leaves, roots, barks	The methanol extracts (1 g/ml) had a ZOI of 16.0 mm compared to (26.0 mm) gentamycin (10 *μ*g/ml) against *B*. *subtilis*	Disk diffusion method	Kisii South	The acute oral median dose (LD_50_) of the root bark extract was >6750 mg/kg body weight. Plant is of relatively low toxicity	[30, 95]
			The organic crude extract exhibited the good inhibition against *B*. *cereus* at 200 and 100 mg/ml concentrations with a ZOI of 13.87 mm and 12.167 mm, respectively, compared to gentamycin 15 mm	Disc diffusion technique	Msambweni District		[74]
			The organic extract exhibited mean inhibition zone values of 24.33 ± 0.33 mm against MRSA compared to streptomycin 39.67 ± 1.76 mm	Agar well diffusion method	Msambweni Kwale County		[81]
*Lantana camara* L. (Verbenaceae)	Skin rashes, boils	Leaves	The methanol leaf extract (1 g/ml) had a ZOI of 17.0 mm compared to gentamycin (10 *μ*g/ml) against *S. aureus*	Agar well diffusion	Bomet District	For short-term use, the extract exhibited very low toxicity, while long-term exposure results in liver and kidneys. the root extract was the most toxic part	[46, 96]
		leaves	The organic leaf extracts MICs and MBCs of 37.5 mg/mL against both *S. aureus* and *P. aeruginosa*	Broth dilution technique	Around Lake Victoria Region		[36]
*Mangifera indica L.* (Anacardiaceae)	Burns, scalds, sores, abscesses, food	Leaves, fruits	The methanol extracts (1 g/ml) had ZOI (18.5 mm) compared to gentamycin 10 *μ*g/ml (19 mm) against *S. aureus*, ZOI (13.0 mm) compared to gentamycin (20 mm) against *E. coli*, ZOI (17 mm) compared to 10 *μ*g/ml gentamycin (18.5 mm) against *P. aeruginosa*	Agar well diffusion	Bomet District	The oral or dermal administration of the extract showed no lethality at the limit doses of 2,000 mg/kg body weight, and no adverse effects were found	[46, 97]
			The methanol extract exhibited a ZOI of 2.07 ± 0.15) cm *against S. aureus* compared to norfloxacin at 10 *μ*g 2.95 cm and ZOI 1.93 ± 0.09) compared to Norfloxacin at 10 *μ*g 2.95 cm against *E. coli*	Disc diffusion method	Makueni and Embu		[98]
*Terminalia brownii* Fresen (Combretaceae)	Diarrhea, ulcers, and sexually transmitted diseases	Bark, leaves, roots	The ethanol extract had ZOI (mm) of 9.2 ± 0.3 compared to 6.8 ± 0.4 (gentamycin) and 6.6 ± 0.2 (erythromycin) against *S. pneumonia*e	Disk diffusion method	Eastern Kenya	The roots and stem bark extracts exhibited mild cytotoxic activity with LC_50_ values ranging from 113.75 to 4356.76 and 36.12 to 1458.81 *μ*g/ml	[18, 99]
			The aqueous leaf extract (1 g/ml) had a ZOI of 18.0 ± 0.8 mm compared to (18.0 ± 0.1) streptomycin (25 *μ*g/ml) against S*. aureus*, ZOI (11.7 ± 0.5 mm) compared to (16.0 ± 0.2) streptomycin (25 *μ*g/ml) against *E. coli*, ZOI (12.8 ± 1.0 mm) compared to (15.0 ± 0.3 mm) streptomycin 25 *μ*g/ml against *B*. *subtilis*	Disk diffusion method	Mbeere, and Embu-Eastern Province		[48]
*Markhamia lutea* (Benth.) K. Schum. (Bignoniaceae)	Eye infection	Bark	The chloroform extracts had a ZOI of 22.82 mm against E.coli, 18.79 mm against *S. aureus* and 17.94 mm against *P. aeruginosa* compared to gentamycin 22.27 mm, 22.52 mm, and 20.17 mm, respectively	Agar well diffusion	Emuhaya Sub-county, Western Kenya	Not reported	[100]
*Asparagus setaceous Kunth* Jessop (Asparagaceae)	Syphilis, gonorrhea	Aerial parts, roots	The MIC values for ethanolic aerial part extract ranged from 3.2 mg/ml for *S. aureus*, 6.25 mg/ml for *E. coli,* and 25 mg/ml for *B*. *subtilis*, *P. aeruginosa*, and *S. faecalis,* while for the ethanolic root extracts of the same plant, MIC ranged from 6.25 mg/ml for *S. aureus* and *B*. *subtilis* to 25 mg/ml for *E. coli*, *P. aeruginosa*, and *S. faecalis*	Broth dilution	Gatundu	Not reported	[101]
*Caesalpinia volkensii Harm* (Caesalpiniaceae)	Bronchitis , pneumonia	Leaves	The MIC values for the ethanolic leaf extract was 6.25 mg/ml for *S. aureus*, 12.5 mg/ml for *B*. *subtilis* and 25 mg/ml for *E. coli* and *P. aeruginosa*	Broth dilution	Gatundu	The organic extract had a median lethal dose of >2000 mg/kg body weight, hence is safe	[101, 102]
*Thylachium africanum Lour.* (Capparaceae)	Diarrhea	Bark	The methanol extract exhibited a ZOI of 18.66 mm against *S. aureus* compared to amoxicillin 21.3 mm, ZOI of 23.33 mm against *P. aeruginosa* compared to amoxicillin 17.5 mm, ZOI of 15 mm against *E. coli* compared to amoxicillin 23.6 mm	Agar disk diffusion technique	Namunyak, Wamba Division, Samburu District	Not reported	[79]
*Alectra sessiliflora* (Vahl) Kuntze (Scrophulariaceae)	Diarrhea, sexually transmitted infections, wounds	Whole plant	The methanol extract 50 mg/ml exhibited ZOI of 15.46 mm against *S. aureus* compared to chloramphenicol 19.23 mm, ZOI of 10.72 mm against *P. aeruginosa* compared to chloramphenicol 19.22 mm, ZOI of 9.76 mm against *E. coli* compared to chloramphenicol 19.10 mm	Disk diffusion method	Vihiga county	Not reported	[103]
*Teclea nobilis* (Rutaceae)	Colds and chest problems	Leaves	The DCM extract exhibited a ZOI of 10 mm against *S. aureus* compared to gentamycin 13 mm and ampicillin 14 mm	Disk diffusion method	Siroch, Keiyo Sub-county, Elgeyo-Marakwet County	Not reported	[104]
*Ochna thomasiana* (Ochnaceae)	Microbial infection	Root, stem barks	The methanolic extract was found effective against *S. aureus* and *B*. *subtilis*, which gave ZOI of 15 mm and 20 mm, respectively, compared to tetracycline 20 mm and 18 mm	Disc diffusion method	Arabuko-Sokoke, forest in Malindi district, Kilifi County	Not reported	[105]
*Cinnamomum cassia* Presl. (Lauraceae)	Food poisoning, flavoring	Fruits	The ethanolic extract of cinnamon was effective against *E. coli* by with a ZOI of 27 mm and MIC of 0.12 *μ*g/ml	Agar well diffusion method/broth dilution	Kenyatta University	Not reported	[66]
*Bidens pilosa L.* (Asteraceae)	Stomach upsets	Leaves	The stem bark DMSO extract exhibited ZOI of 12 ± 0.1 mm compared to Flucloxacillin 14 ± 0.7 mm against *E. coli*	Agar well diffusion method	Narok	Extract showed no adverse effects in mice and chickens at a dose of 5% or less of food	[38, 106]
*Acacia lahai Stead. & HochsLei Benth* (*Fabaceae*)	Skin eruptions	Barks	The methanol extract (200 mg/ml) had a ZOI of 15.00 ± 0.00 mm against *B*. *cereus* compared to Gentamicin (40 ug/ml) 15.83 ± 0.76 mm, ZOI of 11.33 ± 0.29 mm against MRSA compared to Gentamicin (40 *μ*g/ml) 15.67 ± 1.04 mm. The acetone extract (200 mg/ml) had a ZOI of 10.33 ± 0.58 mm against *P. aeruginosa* compared to Gentamicin (40 *μ*g/ml) 15.12 ± 0.63 mm	Disk diffusion method	Mosonik hill, Sotik Sub-county, Bomet County	Not reported	[107]
*Bridelia micrantha* (Hochst.) Baill. (Euphorbiaceae)	Stomachache, diarrhea in children	Leaves	The methanol extract 100 mg/mL exhibited ZOI of 19 mm and 13 mm against *S. aureus* and *S. typhi*	Disc diffusion method	Kilifi District	The extract has a wide margin of safety for oral use at doses below 2000 mg/kg	[49, 108]
*Grewia plagiophylla* K. Schum. (Malvaceae)	Dysentery, typhoid	Leaves	The methanol extract 100 mg/mL exhibited ZOI of 20 mm and 17 mm against *S. aureus* and *S*. *typhi*	Disc diffusion method	Kilifi District	Not reported	[49]
*Vigna subterranea* (*L.*) (Fabaceae)	Traditional food	Nuts	The MIC values for organic extract ranged from *E. coli*—7.72 ± 0.35 *μ*g/ml, *S. aureus*—12.5 ± 0.32 *μ*g/ml, and *P. aeruginosa*—7.95 ± 0.10 *μ*g/ml. At 100 *μ*g/ml, *E. coli*, *S. aureus,* and *P. aeruginosa* showed a ZOI of 27 ± 0.74 mm, 25.3 ± 0.40 mm, and 25.1 ± 0.24 mm, respectively, compared to those of ceftriaxone, which were 37.0 ± 0.5, 41.3 ± 0.9, and 42.3 ± 0.9 mm	Disc diffusion method	Bungoma county	Not reported	[109]
*Citrus limon* (*L.*) *Osbeck* (Rutaceae)	Sore throat, chest pain	Rhizomes	Lemon juice inhibited the growth of *S. typhi* with a ZOI of 11.0 mm and ZOI 11. 0 mm against *P. aeruginosa* compared to chloramphenicol 20.0 ± 0.0 mm	Disc diffusion test	Githurai market, Nairobi	The juice is considered non–toxic and extremely safe for consumption even at above 80% concentration	[64, 110]
*Ziziphus abyssinica* Hochst (Rhamnaceae)	Meat preservative	Fruit paste	The methanolic extract gave ZOI of 24 mm, and 20 mm against *E. coli* and *S. aureus,* respectively, compared to chloramphenicol 16 mm and 18	Disc diffusion test	Chepararia and Kongelai subcounties of West Pokot county	The acute toxicity (LD_50_) of the leaf extracts was found to be greater than 5000 mg/kg and is considered relatively safe for use	[94, 111]
*Mentha spicata* L. (Lamiaceae)	Common cold	Leaves	The ZOI in *S. aureus* varied from 16 ± 0.02 mm in replicate 2 to 18 ± 0.01 mm in replicate 1, *E. coli* (13 ± 0.02 mm in replicate 2 to 15 ± 0.02 mm in replicate 1), and in *K. pneumoniae* (20 ± 0.01 mm in replicate 3 to 20 ± 0.02 mm in replicates 1 and 2)	Agar well diffusion method	Egerton University	The LC_50_ value was 1701 g/ml in brine shrimp lethality assay, indicating that the plant extract is nontoxic	[112, 113]
*Indigofera lupatana Baker F.* (Leguminosae)	Cough, diarrhea, and gonorrhea	Roots	The organic extract showed a highest activity against *B*. *subtilis* (28.5 ± 0.3 mm), *S. aureus* (22.6 ± 1.0 mm), *B*. *cereus* (22.0 ± 0.3 mm), *E. coli* (21.7 ± 0.7 mm), *P. aeruginosa* (21.5 ± 0.9 mm), *S. typhimurium* (17.3 ± 0.3 mm), *K. pneumoniae* (15.3 ± 0.4 mm), and *P. mirabilis* (12.3 ± 0.5 mm)	Disc diffusion assay	Mbeere District, in the Eastern Province of Kenya	The extract had an LC_50_ value greater than 1000 *μ*g/ml which is an indication that they are all nontoxic	[114]
*Momordica* charantia L. (Cucurbitaceae)	Diabetes	Fruit	The extracts exhibited a ZOI of 10.66 mm against *S. aureus* compared to amoxicillin 21.03 mm and MIC and MBC of 37.5 mg/mL , ZOI of 9.33 mm MIC and MBC of 37.5 mg/mL against *P. aeruginosa*	Agar disc diffusion (DD) method/broth dilution technique	Lake Victoria Region	The LD_50_ of the ethanolic extract is considered safe to be consumed below 2000 mg/kg	[36, 115]
*Blighia unijugata* Bak (Sapindaceae)	Tonic, anthelminthic	Roots, pods, and leaves	The methanol and chloroform extracts together with the pure compound, friedelin, were active against *S. aureus* with zones of inhibition of 18.0, 22.0, and 10.0 mm, respectively. Gentamicin (10 *μ*g/rnl) had a ZOI of 26.0 mm against *S. aureus*	Disc diffusion assay	Kiangwachi, Kirinyaga District	The extract has the LD_50_ of 5.628 ± 0.29 g/kg b. wt	[116, 117]
*Moringa oleifera Lam.* (Moringaceae)	Antioxidant, spasms	Seeds, stem	The water extracts showed activity against *S. aureus* with MIC values ranging from 6.25 to 50 mg/ml	Broth microdilution technique		*Moringa oleifera* is genotoxic at supra-supplementation levels of 3000 mg/kg b.wt. However, intake is safe at levels ≤ 1000 mg /kg b.wt	[118, 119]
*Maesa lanceolata* Forssk (Myrsinaceae)	Bacterial infections	Roots, leaves, and stem bark	The stem bark extract exhibited a ZOI of 20.70 ± 0.6 mm against *S. aureus* compared to gentamycin (1.0 *μ*g/disc), 14.30 ± 0.6 and ZOI of 13.00 ± 1.0 mm against *P. aeruginosa* compared to gentamycin (1.0 *μ*g/disc) 16.00 ± 1.0 mm	Disc diffusion method	Elgeyo Marakwet county	DCM extracts of stem bark and leaves were lowly toxic. No mortality was observed within 24 hours	[120]
*Satureja biflora* Buch-Ham (Lamiaceae)	Antimicrobial	Leaves	The essential oil exhibited a ZOI of (31 ± 0.5 mm), MIC 125 mg/mL against *S*. *typhi* and (24 ± 02 mm), 93.8 mg/mL against *S. aureus* compared to chloramphenicol 10 ± 1.0 mm, MIC 25 mg/mL against *S*. *typhi* and 24 ± 1.0 mm, MIC 31 mg/mL against *S. aureus*	Agar disc diffusion method/broth dilution	Botanical garden of Egerton university	Not reported	[86]
*Lannea schweinfurthii* (*Engl.*) *Engl* (Anacardiaceae)	Bacterial infections		An isolated compound epicatechin had zone diameter of growth inhibition of crude extract was (15.05 mm) against *S. aureus* and (14.02 mm) against *B*. *subtilis* compared to tetraycline 18.02 mm against *S. aureus* and *B. subtilis*	Disc diffusion method	Bondo, Siaya County	Not reported	[121]
*Annanus comosus* (Bromeliaceae)	Indigestion	Fruits	The MIC of nanoencapsulated bromelain against *Enterobacter* spp., *Citrobacter* spp., *Serratia* spp., and coagulase-negative *Staphylococci* was 25 *μ*g/ml, while that of *E. coli* was 50 *μ*g/ml. The MIC of nanoencapsulated bromelain against *Klebsiella* spp. and *S. aureus* was 200 *μ*g/ml. Bromelain was effective against gram-positive and gram-negative bacteria. Streptomycin had a MIC of 22.2 *μ*g/ml	Agar well diffusion method/broth microdilution method	Thika Town	Leaf extract is nontoxic	[122, 123]
*Helichrysum forskahlii* (Asteraceae)	Cough	Whole plant	*H. forskahlii* had the highest inhibition zone against MRSA of 19.5 and 18.5 mm in agar well and agar disk diffusion respectively. Chloramphenicol had ZOI 24 mm	Disc diffusion method/agar well diffusion method	Losho, Narok County	The brine shrimp lethality test found the plant to be highly toxic with a lethal concentration of 0.009 mg/ml	[124]
*Citrullus lanatus* (Cucurbitaceae)	Food	Fruit	The MIC value of the nanoparticles was 45.00 ± 0.01 mg/ml for *S. typhi* and 38.50 ± 0.00 mg/ml for E.coli, while the MBC value was 60.00 ± 0.05 mg/ml for *S. typhi* and 50.00 ± 0.00 mg/ml for *E. coli*	Disc diffusion method	Wakulima Market, Muthurwa Market, and Githurai Market within Nairobi county	LD_50_ of EECLS was greater than 2000 mg/kg BW and the no observed adverse effect level (NOAEL) of EECLS was at a dose of 1000 mg/kg in rats	[125, 126]
*Hyptis spicigera* (Lamiaceae)	Stomach ache, pulmonary troubles	Leaves	The methanolic extract (1 g/ml) had a ZOI of 19.3 mm and 19.0 mm against *S. aureus* and *S. typhi,* respectively, compared to amoxicillin 23.0 mm and 21.3 mm. The methanolic extract had an MIC of 37.5 mg/ml against *S. aureus* and *S. typhi* compared to 18.75 mg/ml for amoxicillin	Agar disc diffusion method	Marera Region in Central Province	Not reported	[79]
*Crotalaria quartiniana* (Fabaceae)	Diarrhoea	Leaves	The methanol extract (1 g/ml) had a ZOI of 21.0 mm, 19.3, 21.0, 20.7, 18.7, and 20.7 against *S*. *aureus*, *S*. *typhi*, *E*. *coli*, *P*. *aeruginosa*, and *K. pneumoniae,* respectively, compared to amoxicillin 23.0, 21.3, 20.2, 17.3, and 17. 66 and an MIC of 37.5 mg/ml against *S. aureus*, *S*. *typhi*, *E*. *coli*, *P. aeruginosa*, and *K. pneumoniae* compared to 18.75 mg/ml for amoxicillin	Agar disc diffusion method/broth dilution	Tatu region in central province	Not reported	[79]
*Eurphobia hirta*	Diarrhea, asthma	Whole plant	The methanol extract (1 g/ml) had a ZOI of 21.0 mm, 18.66, 19.66, 16.33, 16.33, and 14.33 against *S*. *aureus*, *S. typhi*, *E*. *coli*, *P*. *aeruginosa*, and *K*. *pneumoniae,* respectively, compared to amoxicillin 23.0, 21.3, 20.2, 17.3, and 17. 66 and MIC of 18.75 mg/ml against *S*. *aureus*, *S. typhi*, *E*. *coli*, *P*. *aeruginosa*, and *K*. *pneumoniae* compared to 18.75 mg/ml for amoxicillin	Agar disc diffusion method/broth dilution	Twiga Region in Central Province	The LD_50_ of this plant is more than 5000 mg/kg	[79, 127]
*Lippia kituiensis* (Verbenaceae)	Diarrhea, chest problems	Leaves	The methanol extract (1 g/ml) had a ZOI of 23.3 mm and 17.6 mm, against *S. aureus* and *P. aeruginosa,* respectively, compared to amoxicillin 23.0, and 17.3. The methanol extract (1 g/ml) had an MIC of 37.5 mg/ml against *S. aureus*	Agar disc diffusion method/broth dilution	Marera Region in Central Province	Not reported	[79]
*Eurphobia scarlatina* (*Euphorbiaceae*)	Stomach ache, common cold, TB	Stem	The extract exhibited zero GUs at 0.5 mg/ml against *M. kansasii* and *M. tuberculosis* compared to Isoniazid that had zero GUs at 0.5 mg/ml	BACTEC mgIT™ 960 system	Various conservancies in Samburu	Not reported	[36]
*Acacia horrida.* (Fabaceae)	Diarrhoea, TB	Barks	*A*. *horrida* had appreciable inhibition (257 GUs) against *M. tuberculosis* (198 GUs) at the concentration of 0.5 mg/ml compared to Isoniazid that had zero GUs at 0.5 mg/m	BACTEC mgIT™ 960 system	Various conservancies in Samburu	Not reported	[36]
*Phyllanthus urinaria Linn* (*Phyllanthaceae*)	Dysentery, diarrhea, stomach ache	Leaves, roots	MIC and MBC of 18.75 mg/ml and 37.50 mg/ml, respectively, against E. coli	Broth dilution	Transmara West	Not reported	[51]
*Rhamnus prinoides L'He'r* (Rhamnaceae)	Typhoid, stomach ache	Stem, roots	The extract inhibited *E. coli* with MIC and MBC of 9.37 mg/ml	Broth dilution	Transmara west	Rhamnus prinoides was nontoxic to brine shrimp	[51, 128]
*Tetradenia riparia* (Lamiaceae)	Respiratory problems, stomach ache, diarrhea, antiseptic	Roots, stem	The organic extract inhibited *S. epidermidis* with a ZOI of 27.67 ± 0.333 mm compared to penicillin 26.67 ± 0.333 and E*. coli* with a ZOI of 13.33 ± 0.333 mm compared to penicillin 31.33 ± 0.333	Disc diffusion	Natural forest around the University of Eastern Africa, Baraton	Toxic effect recorded for root and fruit extracts but not for leaf or stem extracts. In mice at dose 1.0 g/kg	[61, 129]
*Kigelia africana Lam and Benth* (*Bignoniaceae*)	Laxative, gonorrhea, tuberculosis, diarrhea	Fruits, barks	The methanolic extract had a ZOI of 11.3 mm compared to (19 mm) gentamycin against *S. aureus* and ZOI (10 mm) compared to (9 mm) chloramphenicol against MRSA. The MIC values of acetone extracts were 6.25 mg/ml against MRSA	Disc diffusion	Kaptumo Division, Nandi	Not reported	[130]
*Conyza sumatrensis* (Asteraceae)	Pimples	Leaves/roots	The methanolic extract had a ZOI of 26.85 mm compared to (13.67 mm) chloramphenicol against *E. coli* and ZOI (27 mm) compared to (15.8 mm) chloramphenicol against *B*. *pumulus*	Agar diffusion assay method	Rarieda, Bondo district, of Nyanza province in Kenya	Experiments indicate the methanol extract to be safe even at high and repeated doses in pre-clinical studies	[131, 132]
*Piliostigma thonningii* (Fabaceae)	Cough, colds, chest pains, stomachache, wounds	Stem bark	The methanolic extract had MIC (3.125 mg/ml) compared to (>1 mg/ml for antibiotic standards) against *S. aureus*, MIC (31.25 mg/ml) compared to (>1 mg/ml for antibiotic standards) against and *E. coli* and MIC (15.625 mg/ml) *against P. aeruginosa*	Broth dilution method	Meru central district	Plant extracts had LD_50_ values >2000 mg/kg bw and were hence deemed to be nontoxic	[40, 133]
*Erythrina abyssinica* (Fabaceae)	Anthrax, syphilis, gonorrhea, burns, body swellings	Root bark	The methanolic extract had an MIC of 3.125 mg/ml compared to >1 mg/ml for antibiotic standards against *S. aureus*, MIC (250 mg/ml) compared to (>1 mg/ml for antibiotic standards) against and *E. coli* and MIC (125 mg/ml) against *P. aeruginosa*	Test tube method	Meru Central District	The extracts are not toxic to the human cell	[40, 134]
*Rynchosia minima DC.* (Fabaceae)	Swelling	Roots	The methanolic extract had a ZOI of 11.5 mm compared to 15 mm gentamycin against *S. aureus*	Disk diffusion technique	Central Kenya	Not reported	[135]
*Entada abysinnica* (Fabaceae)	Gastrointestinal bacterial infections, bronchitis	Leaves	The methanol leaf extract (100 mg/ml) had ZOI (10. 33 mm) compared to (16.0 mm) zeftazidime against *S. typhi*	Disk diffusion technique	Bondo (Sakwa) in western Kenya	Not reported	[36]
*Withania somnifera* (Solanaceae)	Microbial infections, cholesterol-lowering	Leaves, roots	The dichloromethane extract (100 mg/ml) had a ZOI of 16.0 mm compared to (18.0 mm) chloramphenicol against *S. aureus*, ZOI (14.0 mm) compared to (24.0 mm) chloramphenicol against MRSA and MIC of 6.25 mg/ml against *S. aureus* and 12.5 mg/ml against MRSA	Disc diffusion test/broth dilution	Ngong forest	The extract is relatively safe for use even in dose levels exceeding 200 *μ*g/ml	[59]
*Thalictrum rhynchocarpum* (Ranunculaceae)	Stomach discomfort and bacterial infections	Roots, bark	The root extract had an MIC of 21.5 mg/ml against *B*. *subtilis* compared to ciprofloxacin 21.5 mg/ml	Broth dilution	Ngong Forest	Not reported	[136]
*Hugonia castaneifolia* (Linaceae)	Intestinal worms	Roots	The dichloromethane stem bark extract (100 mg/ml) was active against *S. aureus* (MIC 0.0008 mg/ml, respectively). Hexane stem bark extracts were active against *S. aureus* at 0.0031 mg/ml gentamicin and had an MIC of 0.5 mg/ml	Broth dilution	Coast Province of Kenya	Not reported	[29]
*Tabernaemontana stapfiana Britten* (Apocynaceae)	STIs and respiratory-tract infections	Stem bark, root bark, fruits and leaves	The ethanolic root extract (100 mg/ml) had a ZOI of 18.0 mm compared to (22.0 mm) chloramphenicol against MRSA and multiple drug-resistant *S. aureus* (MDRS) and MIC of 3.9 *μ*g/ml	Disc diffusion test/broth dilution	Kaptagat Forest in Keiyo District	Not reported	[137]
*Rhus vulgaris* (*Anacardiaceae*)	GIT disorders	Leaves, bark	The methanol extract of *Rhus vulgaris* showed significant antimicrobial activity against MRSA (12.00 ± 0.00 mm; MIC of 0.391 mg/ml; minimum bactericidal concentration of 1.563 mg/ml). Compared to (6.00 ± 0.00 mm) sulfamethoxazole/trimethoprim (23.7:1.25 *μ*g). The methanol extract showed significant antimicrobial activity against *S. aureus* (19.50 ± 0.71 mm; MIC of 0.391 mg/ml; compared to (24.19 ± 3.60 mm) sulfamethoxazole/trimethoprim (23.7:1.25 *μ*g)	Disc diffusion assay/minimum inhibitory concentration assay	Mwala Sub-County, Machakos County	There were no observable adverse effects from oral administration of the extracts (acute oral toxicity testing) at concentrations of 50 mg/kg, 300 mg/kg, and 2000 mg/kg	[138]
*Zanthoxylum paracanthum Kokwaro* (*Rutaceae*)	Diarrhoea	Root bark	The CH_2_Cl_2_/CH_3_OH (1 : 1) extract from the root bark had MIC values of 3.91, 1.95, 0.98, and 7.81 *μ*g/mL, against MRSA, *E. coli*, *S. aureus* compared to 0.98, 0.49, and 0.98 *μ*g/ml of omacillin	Minimum inhibitory concentration assay	Mrima Hills, Kwale County in Kenya	Not reported	[139]
*Centella asiatica* (Apiaceae)	Bacterial infections, diarrhea, skin lesions, psoriasis, keloids	Leaves	Organic crude extract of the leaf showed the highest activity ZOI of 16.33 ± 0.33 mm against *E. coli* compared to tetracycline 26.67 ± 0.33 mm	Disc diffusion method	Kisii County	The lethal dose and no observable adverse effect level were 2000 mg/kg and 1000 mg/kg	[32, 140]
*Aloe vera* (Asphodelaceae)	Blood purifier, malaria, skin disease, diabetes	Leaves	The organic extract exhibited a ZOI of 17 ± 2 − 19 ± 2 mm against *S. aureus*, (18 ± 2 − 20 ± 1 mm against *B*. *subtilis*), (17 ± 1 − 19 ± 3 mm) against *K. pneumoniae*, (16 ± 1 − 20 ± 3 mm) against *E. coli*	Agar diffusion method	Department of biological sciences, Egerton university	Not reported	[141]
*Aloe volkensii* (Asphodelaceae)	Laxative, burns, wounds and sores	Leaves	The organic extract exhibited activity against *S. aureus* (19 ± 1 − 20 ± 2 mm), *B*. *subtilis* (17 ± 2 − 21 ± 3 mm), *K. pneumoniae* (18 ± 2 − 19 ± 1 mm), *E. coli* (18 ± 2 − 19 ± 3 mm	Agar diffusion method	Department of Biological Sciences, Egerton University	Not reported	[141]
*Senna spectabilis* (Fabaceae)	Laxatives	Leaves, pods	The organic leaf extract (100 mg/ml) had a ZOI of 9.6 ± 0.6 compared to chloramphenicol (11.7 ± 2.3) against *S. typhi*	Agar diffusion method	Mbeere North District, Embu County	Not reported	[142]
*Maytenus putterlickioides* (Celastraceae)	Malaria, emmenagogue, aphrodisiac	Roots	The methanol root extract (100 mg/ml) had a ZOI of 9.2 ± 1.1 compared to chloramphenicol (11.7 ± 2.3) against *S. typhi*	Agar diffusion method	Mbeere North District, Embu county	Not reported	[142]
*Olinia usambarensis* (Oliniaceae)	Malaria, abscess, cough, measles	Bark, roots, leaves	The methanol leaf extract (100 mg/ml) had ZOI (12.2 ± 0.8) compared to chloramphenicol (11.7 ± 2.3) against *S. typhi*	Agar diffusion method	Mbeere North District, Embu County	Not reported	[142]
*Crotalaria goodformis Vatke.* (Fabaceae)			The aqueous extract (1 g/ml) had a ZOI of 14.8 ± 0.2 mm compared to (18.0 ± 0.1) streptomycin (25 *μ*g/ml) against *S. aureus*	Disk diffusion method	Mbeere, and Embu-Eastern Province	Not reported	[48]
*Prosopis juliflora* (*Sw.*) *DC* (Fabaceae)	Open wounds and dermatological ailments.	Leaves	The ethanolic leaf extract (100 mg/ml) had a ZOI of 20.00 ± 1.00 mm compared to (19.0) erythromycin (15 *μ*g/ml) and (30.0 mm) chloramphenicol (30 *μ*g/ml) against *E. coli*, ZOI (15.33 ± 0.58 mm) compared to (11.0) erythromycin (15 *μ*g/ml) and (22.0 mm) chloramphenicol (30 *μ*g/ml) against *P. aeruginosa*	Disk diffusion method	Endao, Marigat District, in Baringo County	The pods are toxic, mainly for cattle and goats, and have piperidine alkaloids and can cause neurotoxicity	[143, 144]
*Osyris abyssinica* (Santalaceae)	Dysentery, typhoid	Roots	The aqueous root extract (1 g/ml) had ZOI (15.2 ± 0.7 mm) compared to (18.0 ± 0.1) streptomycin (25 *μ*g/ml) against *S. aureus*, ZOI (14.8 ± 0.3 mm) compared to (16.0 ± 0.2) streptomycin (25 *μ*g/ml) against *E. coli*, ZOI (15.5 ± 0.5 mm) compared to (15.0 ± 0.3 mm) streptomycin 25 *μ*g/ml against *B*. *subtilis*	Disk diffusion method	Mbeere, and Embu-eastern province	Not reported	[48]
*Abrus precatorius* (Fabaceae)	Gonorrhea, coughs in children	Leaves, roots	The aqueous leaf extract (1 g/ml) had a ZOI of 15.7 ± 0.5 mm compared to (18.0 ± 0.1) streptomycin (25 *μ*g/ml) against *S*. *aureus*. The aqueous bark extract (1 g/ml) had a ZOI of 7.2 ± 0.8 mm compared to (16.0 ± 0.2) streptomycin (25 *μ*g/ml) against *E. coli*, ZOI (15.5 ± 0.5 mm) compared to (10.7 ± 1.2 mm) streptomycin 25 *μ*g/ml against *B*. *subtilis*	Disk diffusion method	Mbeere, and Embu-Eastern Province	It contains abrin, a toxalbumin that inhibits protein synthesis causing cell death. especially seeds	[48, 145]
*Ormocarpum trichocarpum* (Fabaceae)	Bone setting	Roots	The methanol extract (1 g/ml) had a ZOI of 15.5 mm compared to (26.0 mm) gentamycin (10 *μ*g/ml) against *B*. *subtilis*	Disk diffusion method	Kisii South	Not reported	[30]
*Psidium guajava* (Myrtaceae)	Wounds, ulcers, cholera	Leaves	The methanol extracts (1 g/ml) had a ZOI of 19.7 mm compared to gentamycin (10 *μ*g/ml) against *S. aureus*, ZOI (16.0 mm) compared to gentamycin (10 *μ*g/ml) 19 mm against *E. coli* and, ZOI (16 mm) compared to 10 *μ*g/ml gentamycin (17 mm) against *P. aeruginosa*	Agar well diffusion	Bomet District	The median lethal dose (LD_50_) of bark extract is greater than 5000 mg/kg body weight	[46, 145]
*Cyathula polycephala* (Amaranthaceae)	Diabetes, skin infections, pneumonia	Stem barks	The methanol extract 100 mg/ml had a ZOI of 14.2 mm compared to 30 *μ*g/ml gentamycin (19.0 mm) against *S. aureus*, ZOI (16.0 mm) compared to 30 *μ*g/ml gentamycin (9.0 mm) against MRSA, ZOI (10.0 mm) compared to 30 *μ*g/ml gentamycin (21.0 mm) against *P. aeruginosa*	Disk diffusion method	Bomet District	The methanol extract was very safe with a CC_50_ of 100%, while water extract were toxic with CC_50_ of 23.75% and 31.56% as compared to the positive control Chloroquine with CC_50_ of 25.28 and 51.94% at concentration 1000 and 100 mg mL-1	[29]
*Blumea axillaris* (Lam.) DC. (Asteraceae)	Skin disease	Aerial parts	Methanol extracts had a ZOI 16.41 ± 0.31 compared to chloramphenicol (21.7 ± 0.11) against *S. aureus*	Agar well diffusion	Vihiga County, Western Kenya	None reported	[45]
*Chamaecrista mimosoides* (*L.*) *Greene* (Fabaceae)	Respiratory system disorders, dysentery	Aerial parts, roots	The aqueous extracts had a ZOI 30.00 ± 1.46 compared to chloramphenicol (21.7 ± 0.11) against *S. aureus*	Agar well diffusion	Vihiga County, Western Kenya	Not reported	[45]
*Lantana trifolia L.* (Verbenaceae)	Cough and common colds	Aerial parts	The methanol extracts had a ZOI of 20.59 ± 0.92 compared to chloramphenicol (21.7 ± 0.11) against *S. aureus*	Agar well diffusion	Vihiga County, Western Kenya	The ethanol extracts of Lantana trifolia (LC_50_ 32.3 *μ*g/ml exhibited mild toxicity and are safe for short-term use (Moshi et al., 2010)	[45]
*Terminalia kilimandscharica Engl.* (*Combretaceae*)	Cough, sexually transmitted diseases	Barks	The methanolic bark extract had a MIC of 25, 15.6, 37.5, and 150 mg/mL against *S. aureus B. cereus*, *P. aeruginosa,* and *E. coli,* respectively, compared to that of 0, 0, 0.25, 0.25 mg/mL of streptomycin against *S. aureus B. cereus*, *P. aeruginosa.* and *E. coli,* respectively, and benzylpenicillin 0.6 and 0.6 against S. aureus and B. cereus, respectively	Broth dilution	Machakos and Kitui	The methanolic bark extracts had an LC_50_ of <1000 *μ*g/mL, which is considered relatively nontoxic	[39]
*Pentas lanceolata* (Rubiaceae)	Genital and oral thrush	Roots	The ethyl acetate extract (100 mg/ml) had a ZOI of (10.96 ± 0.08 mm) compared to gentamycin (23.88 ± 0.01 mm) against *K. pneumoniae*, ZOI of (12.08 ± 0.26 mm) compared to gentamycin (23.88 ± 0.01 mm) against *Escherichia coli* and ZOI of (11.39 ± 0.6 mm) compared to gentamycin (25.9 ± 0.01 mm) against *S. aureus*	Agar well diffusion	Magadi, Kajiado District of Kenya	Not reported	[44]
*Sericocomposis hildebrandtii* Schinz (Amaranthaceae)	Purgative	Roots	The ethyl acetate root extract (100 mg/ml) had a ZOI of 11.28 ± 0.09 mm compared to gentamycin (0.1 *μ*g/ml), (23.88 ± 0.01 mm) against *K. pneumoniae*, ZOI of (10.33 ± 0.06 mm) compared to gentamycin (0.1 *μ*g/ml) (23.88 ± 0.01) against *E. coli*, and ZOI of (10.66 ± 0.18) compared to gentamycin (0.1 *μ*g/ml) (25.9 ± 0.01) against *S. aureus*	Agar well diffusion	Magadi, Kajiado District of Kenya	Not reported	[44]
*Combretum molle R.Br. ex G.Don* (Combretaceae)	Tooth brush, stomach ache, and dysentery	Stem bark	The ethanolic stem bark extract (0.5 mg) exhibited ZOI (mm) of 7.6 ± 0.24 against *P. aeruginosa*, 15.4 ± 0.3 against *E. coli*, and 2.2 ± 0.4 against *S. aureus* compared to Augmentin 8.0 ± 0.02 against *P. aeruginosa*, 19.0 ± 0.03 against *E. coli* and 17.0 ± 0.02 against *S. aureus*	Disc diffusion method	Mwingi District in Kitui County	For the acute toxicity test, no death and signs of poisoning were observed in the treated groups. In the subacute study, LD_50_ in the rats after intraperitoneal administration was 700 mg/kg	[76, 146]
*Combretum illairii* (Combretaceae)		Roots, stems, leaves	The methanol leaf extract (100 mg/ml) had ZOI of 15.60 mm and 17.00 mm against *S. aureus* and *P. aeruginosa,* respectively, against gentamycin (30 *μ*g/ml) 25.3 mm and 18 mm	Disc diffusion assay	Kilifi District	The stem bark extracts had neither cytotoxicity nor brine shrimp lethality. plant extracts	[29]
*Combretuam tanaense* (Combretaceae)	Skin infections, wounds dressings and ointments	Roots	The methanol root extract (100 mg/ml) had a ZOI of 11.50 ± 0.5 mm compared to ciprofloxacin (0.32 *μ*g/ ml) 14.75 ± 0.25 mm against *S. aureus* and ZOI of 12.25 ± 0.25 mm compared to ciprofloxacin (0.32 *μ*g/ ml) 16.00 ± 0.00 mm against *K. pneumoniae*	Agar well diffusion assay	Mount Kenya University Botanical Garden, Thika	Not reported	[104]
*Vernonia brachycalyx* (Asteraceae)	Antimalarial, emetic	Stem, leaves	The ethanol extract had a ZOI of 9.5 ± 1.2 mm against *S. pneumoniae* compared to 8.2 ± 0.6 (erythromycin) 7.2 ± 0.5 (gentamycin 15 *μ*g)	Disk diffusion method	Eastern Kenya	An isolated compound (16,17-dihydrobrachycalyxolide) displayed high toxicity against human lymphocytes	[18]
*Vernonia amygdalina* (Asteraceae)	Stomach discomfort and bacterial infections	Leaves	The methanol leaf extract (1 g/ml) exhibited a ZOI 17.0 mm compared to gentamycin (10 *μ*g/ml) 19 mm against *S. aureus*	Agar well diffusion	Bomet District	The extract had an LD_50_ of 288.5 mg/kg body weight. It has relative toxicity [147]	[46]
*Vernonia glabra* (*Steetz*) *Oliv*. *& Hiern* (Asteraceae)	Gastrointestinal problems , snake bites	Leaves, roots	The dichloromethane/methanol leaf extract (1 g/ml) exhibited a ZOI of 1.85 cm against *S. aureus* compared to streptomycin with a ZOI of 1.30 cm. dichloromethane/methanol extract of flower showed significant activity only against *S. aureus*, with the lowest MIC of 1.5625 mg/100 *μ*l, compared to streptomycin with a MIC of 6.25 mg/100 *μ*l	Disc diffusion	Machakos	Not reported	[27]
*Vernonia adoensis* (Asteraceae)	Oral health	Stem bark	The methanol stem bark extract (100 mg/ml) exhibited a ZOI of 13.00 ± 0.577 mm against *E. aerogenes*, 11.00 ± 0.577 mm against *S. pyogenes*, 9.67 ± 0.333 mm against *S. epidermidis,* and 9.00 ± 0.577 mm against *E. faecalis*. The acetone stem bark extract (100 mg/ml) exhibited a ZOI of 16.00 ± 0.577 mm against *E. aerogenes*, 11.33 ± 0.882 mm against *S. epidermidis,* and 10.00 ± 0.577 mm against *S. faecalis*. Penicillin (100 mg/ml) exhibited a ZOI of 38.00 ± 0.577 mm against *E. faecalis,* 43.33 ± 0.882 against *S. pyogenes*, 19.33 ± 0.333 against *S. epidermidis,* and 36.33 ± 0.882 against *E. aerogenes*	Disc diffusion method	Natural forests around the University of Eastern Africa, Baraton, Nandi County	Not reported	[148]
*Vernonia hymenolepis* (Asteraceae)	Infections, toothache	Leaves	The MBC and MIC values of aqueous leaf extract was 400 mg/ml against *S. aureus,* while the DCM/methanol leaf extract had MIC and MBC of 400 mg/ml against *P. aeruginosa and E. coli*, and MIC of 100 mg/ml against *S*. *aureus*. Amoxicillin had MIC and MBC of 3.125 mg/ml and 6.25 mg/ml against *E. coli*, respectively	Broth dilution	Trans Nzoia County	Not reported	[149]

## Data Availability

No data were used in the study.
